# Extremely Late In-Stent Thrombosis 12 Years After Implantation of a Drug-Eluting Stent

**DOI:** 10.7759/cureus.9053

**Published:** 2020-07-07

**Authors:** Elsa Sleiman, Rabih Tabet, Boutros Karam, David Ayad, Roman Royzman

**Affiliations:** 1 Internal Medicine, Staten Island University Hospital - Northwell Health, Staten Island, USA; 2 Cardiovascular Medicine, Staten Island University Hospital - Northwell Health, Staten Island, USA; 3 Cardiology, Staten Island University Hospital - Northwell Health, Staten Island, USA; 4 Internal Medicine, Staten Island University Hospital, Staten Island, USA; 5 Cardiology, Staten Island University Hospital, Staten Island, USA

**Keywords:** extremely late stent thrombosis, very late stent thrombosis, drug-eluting stent, st-segment elevation myocardial infarction

## Abstract

Stent thrombosis is one of the most feared complications of percutaneous coronary intervention. Most commonly it occurs within the first few days after the deployment of the stent. Once the stent is completely endothelialized, this complication becomes extremely rare. Few cases of very late stent thrombosis were reported in the literature with the longest interval being around 11 years after the initial intervention. We report here the case of a 78-year-old male patient who presented with acute onset chest pain found to have acute inferior ST-segment elevation myocardial infarction due to thrombotic occlusion of a prior paclitaxel drug-eluting stent placed 12 years prior. This is, to our knowledge, the first case of stent thrombosis occurring after this long duration since stent implantation.

## Introduction

Percutaneous coronary intervention with stent implantation is a successful treatment for patients with obstructive coronary artery disease and has been shown to improve symptoms and reduce mortality [1,2]. Previously, bare metal stents (BMS) were used, and now the majority of recently used stents are drug-eluting stents (DES). First and second-generation DES and BMS present the risk of in-stent thrombosis, with newer second-generations demonstrating the lowest risk of late stent thrombosis [3,4]. This serious complication can occur acutely (within the first 24 hours of stent deployment), subacutely (within 30 days), late (within the first year), or very late (more than a year post deployment). Most patients with stent thrombosis present with ST-segment elevation on electrocardiogram (STEMI). It is caused by a total or near total thrombotic occlusion of the intracoronary stent. Few cases of very long stent thrombosis are reported in the literature with the longest reported period being 11 years [5,6]. We report a patient who presented with a very late stent thrombosis (VLST) that occurred 12 years after implantation. It is unique being the longest duration reported so far in the literature. We will cover in our discussion the possible underlying mechanisms and risk factors, as well as the implications of stent thrombosis on our practice. 

## Case presentation

**Figure 1:**
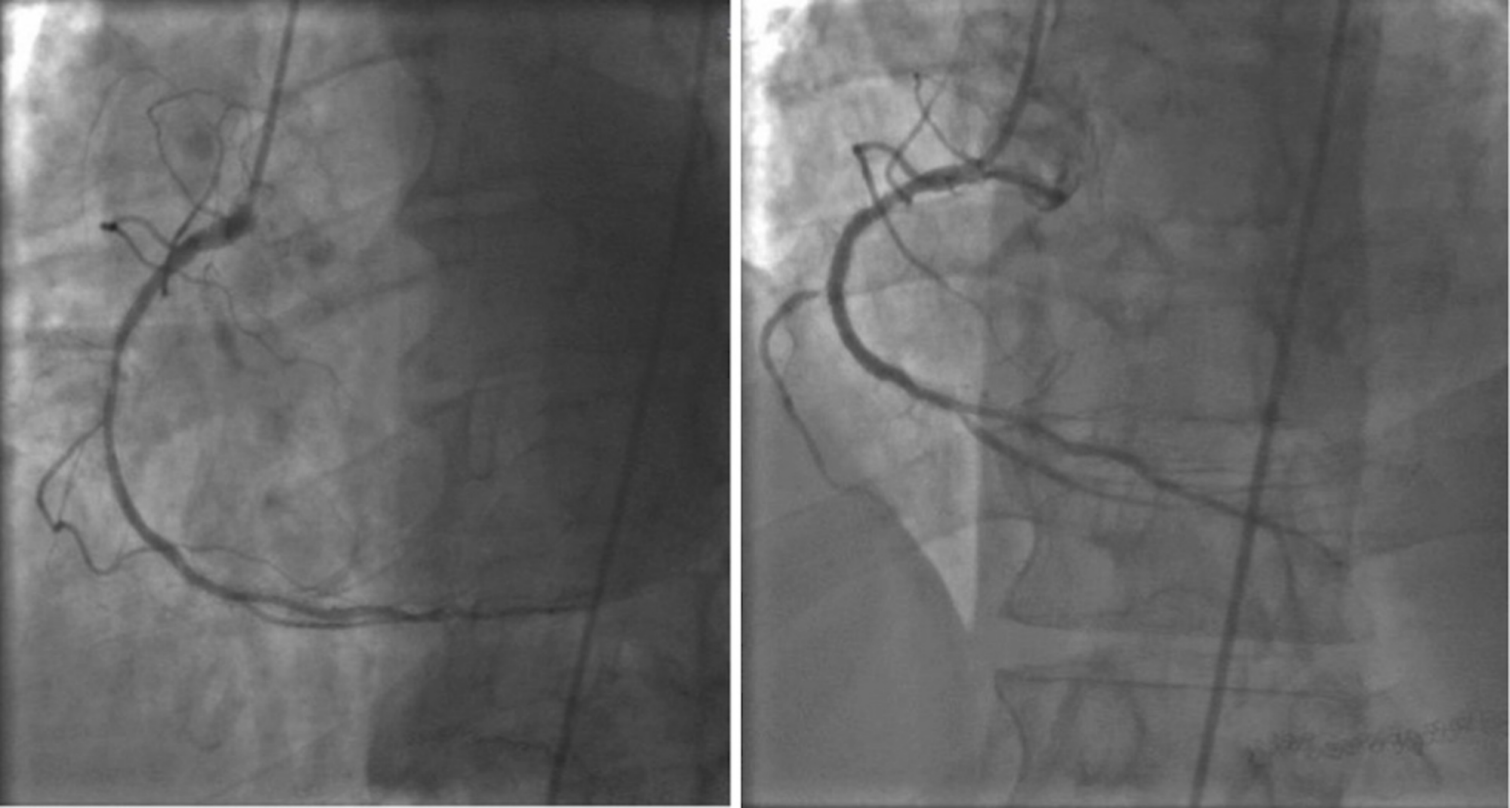
Initial percutaneous angioplasty of the right coronary artery with drug-eluting stenting performed in 2006

A 78-year-old man presented to our emergency department because of acute onset chest pain that started two hours prior to presentation. The pain was retrosternal, pressure-like, moderate in intensity and started upon awakening from sleep. His past medical history is significant for type II diabetes mellitus and pancreatic cancer that was treated with the Whipple procedure 27 years ago. He had coronary artery disease status post percutaneous angioplasty with stenting of the mid right coronary artery (RCA) 12 years ago, and stenting of the proximal circumflex and proximal RCA 17 years ago. A paclitaxel drug-eluting stent (PES) 3.0 x 24 mm was used to stent the mid RCA 12 years ago (Figure 1). 

**Figure 2:**
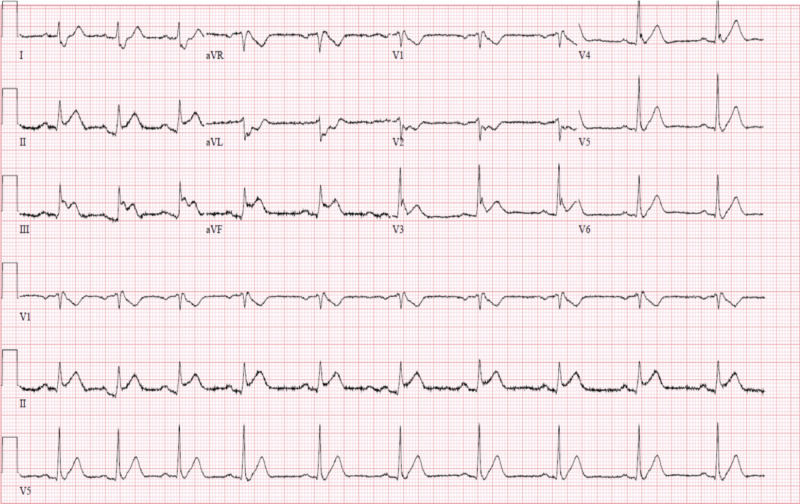
Electrocardiogram upon presentation to the emergency room showing ST-segment elevation in leads II-III-aVF

The patient is a former cigarette smoker, and does not consume alcohol, caffeine, or illicit drugs. At presentation, he was in mild distress, complaining of typical chest pain persistent despite aspirin administration. On physical examination, the patient was noted to be diaphoretic. His heart rate was 60 bpm, and blood pressure was 110/75 mmHg. Electrocardiogram (ECG) showed ST-segment elevation in the inferior leads II, II, and avF (Figure 2).

**Figure 3:**
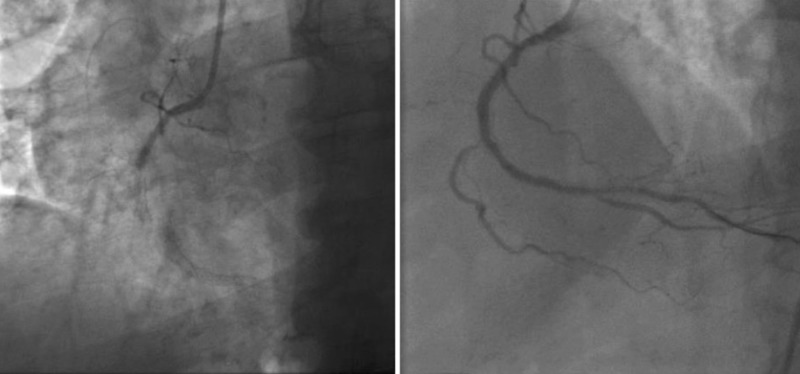
Angiogram of the right coronary artery showing acute in-stent thrombosis followed by percutaneous coronary intervention with complete revascularization of the artery

The patient was loaded with aspirin and clopidogrel and emergently taken to the cardiac catheterization laboratory. Coronary angiography showed thrombotic occlusion of mid RCA DES placed 12 years ago. Immediate percutaneous coronary balloon angioplasty was performed followed by a 3.5 x 16 mm everolimus drug-eluting stent (EES) deployment at a maximum inflation pressure of 14 atm. Following the intervention, excellent angiographic appearance of the artery was obtained with a 0% residual stenosis (Figure 3).

Due to the acuity of the situation and the patient’s unstable hemodynamic status, it was difficult to obtain intravascular images of the lesion and tell with certainty what mechanism led to this event. The patient was stabilized and monitored for the following 24 hours with no further complications, and was then successfully discharged home.

## Discussion

Stent thrombosis occurring at any time is a serious complication carrying a significant risk of death. Although VLST is infrequent, it is being reported more in the literature with DES. According to the academic research consortium, our patient fits the criteria of having a “definite stent thrombosis” even with the absence of intravascular imaging. Angiographic diagnosis is made by identifying a thrombus originating in or within 5 mm of the stent along with the presence of one of the following criteria: acute onset of ischemic symptoms, new ischemic ECG changes, typical rise and fall in cardiac biomarkers, or pathologic confirmation following thrombectomy or by autopsy [7]. Etiology, pathogenesis, and predictive factors have not yet been established due to relatively low prevalence of this condition and its multifactorial nature. The possible mechanisms of thrombosis that have been evaluated through real-time imaging studies using intravascular ultrasound, angioscopy, or optical coherence tomography, and tissue histology are still not fully understood. However, potential mechanisms and explanations of this late in-stent thrombosis include (1) delayed neointimal coverage (uncovered stent struts), (2) stent underexpension, (3) ongoing vessel inflammation, (4) neoatherosclerosis rupture, and (5) late stent malapposition, the latter being the most common [8-10]. Risk factors for stent thrombosis in general are well known, but some of these factors have been specifically associated with VLST. These include smoking history at the time of the stent implantation, presence of thrombus, multivessel disease, type C lesions, longer total stented length, and overlapping stents [11]. Another major factor affecting the risk of stent thrombosis is the type of stent used. Several concerns have emerged about the higher risk of VLST with first-generation DES: paclitaxel drug-eluting stent (PES) and sirolimus drug-eluting stent (SES) [12,13]. Studies comparing PES to newer generation DES showed that EES is associated with a significant reduction of ST at long-term follow-up, and PES having a higher risk of VLST [4,14,15]. 

## Conclusions

As we report the longest case of stent thrombosis so far in the literature, we advocate the need of heightened awareness of these risks especially with first-generation PES and the other risk factors mentioned above. Further studies are needed to better understand the exact pathology behind very late in-stent thrombosis and help preventing them. Until then, we advise physicians to address as much as possible the underlying modifiable risk factors mentioned above. And when available, the use of intravascular imaging pre- and post-stenting is highly encouraged to guide selecting the appropriate stent size and adequate deployment of the stent on a healthy endothelium, and ensuring well apposition and expansion of the stent, particularly when dealing with proximal lesions.
